# Real-World Data Analysis of Patients Affected by Immune-Mediated Thrombotic Thrombocytopenic Purpura in Italy

**DOI:** 10.3390/jcm13051342

**Published:** 2024-02-27

**Authors:** Emanuele Angelucci, Andrea Artoni, Luana Fianchi, Melania Dovizio, Biagio Iacolare, Stefania Saragoni, Luca Degli Esposti

**Affiliations:** 1 U.O. Ematologia e Terapie Cellulari, IRCCS Ospedale Policlinico San Martino, 16132 Genova, Italy; emanuele.angelucci@hsanmartino.it; 2 Fondazione IRCCS Ca’ Granda Ospedale Maggiore Policlinico, Angelo Bianchi Bonomi Hemophilia and Thrombosis Center, 20122 Milano, Italy; andrea.artoni@policlinico.mi.it; 3 Dipartimento Scienze Radiologiche, Radioterapiche ed Ematologiche, Fondazione Policlinico Universitario A. Gemelli, IRCCS, 00168 Roma, Italy; luana.fianchi@policlinicogemelli.it; 4 CliCon S.r.l. Società Benefit, Health, Economics & Outcomes Research, 40137 Bologna, Italy; melania.dovizio@clicon.it (M.D.); biagio.iacolare@clicon.it (B.I.); stefania.saragoni@clicon.it (S.S.)

**Keywords:** immune-mediated thrombotic thrombocytopenic purpura, caplacizumab, epidemiology, mortality, real-world evidence

## Abstract

**Background:** The therapeutic management of immune-mediated thrombotic thrombocytopenic purpura (iTTP) has recently benefited from the introduction of caplacizumab, an agent directed at the inhibition of platelet aggregation. This real-world analysis investigated the epidemiology and the demographic and clinical characteristics of iTTP patients in Italy before and after caplacizumab introduction in 2020. **Methods:** Hospitalized adults with iTTP were included using the administrative databases of healthcare entities covering 17 million residents. Epidemiological estimates of iTTP considered the 3-year period before and after caplacizumab introduction. After stratification by treatment with or without caplacizumab, iTTP patients were characterized for their baseline features. **Results:** The annual incidence before and after 2020 was estimated in the range of 4.3–5.8 cases/million and 3.6–4.6 cases/million, respectively. From 2018 to 2022, 393 patients with iTTP were included, and 42 of them were treated with caplacizumab. Caplacizumab-treated patients showed better clinical outcomes, with tendentially shorter hospital stays and lower mortality rates (no treated patients died at either 1 month or 3 months after caplacizumab treatment initiation, compared to 10.5% and 11.1% mortality rates at 1 and 3 months, respectively, of the untreated ones). **Conclusions:** These findings may suggest that caplacizumab advent provided clinical and survival benefits for patients with iTTP.

## 1. Introduction

Thrombotic thrombocytopenic purpura (TTP) is a rare blood disorder characterized by clotting in small blood vessels that leads to thrombocytopenia and hemolytic anemia [1]. TTP is caused by congenital (cTTP) or by immune-mediated (iTTP) deficiency/absence of von Willebrand factor (VWF)-cleaving protease ADAMTS13 (a disintegrin and metalloproteinase with a thrombospondin type 1 motif member 13), leading to platelet consumption in VWF-platelet aggregates and ultimately microvascular thrombosis [2]. As a result of impaired ADAMTS13 activity, VWF multimers accumulate on the endothelial surface, thus triggering platelet aggregation with subsequent thrombi formation [1]. This detrimental cascade results in end-organ ischemia and damage, with the central nervous system (CNS), heart, and kidneys as the most injured organ systems [1]. If untreated, iTTP can be a fatal disease in approximately 90% of the cases, but therapeutic interventions can dramatically decrease mortality rates to 10–15% [3].

Historically, iTTP has been managed by therapeutic plasma exchange (TPE) [4] and immunosuppressive therapy [5,6,7]. However, in spite of the benefits of TPE in improving platelet count and reducing thrombotic complications in the short term, it does not correct the underlying formation of anti-ADAMTS13 autoantibodies. For this reason, patients who initially respond to TPE may experience a rapid decline in their platelet count after TPE interruption, even if co-treated with immunosuppressive drugs, like corticosteroids and rituximab. This phenomenon, known as “exacerbation”, is due to the persistent antibody-mediated severe ADAMTS13 deficiency [4,5,6,7].

The therapeutic options for iTTP patients have recently been enlarged by the introduction of caplacizumab, a new anti-VWF humanized single-variable domain immunoglobulin (nanobody) able to prevent VWF-platelet aggregation via inhibition of the interaction between ultra-large VWF multimers and platelets [8]. The effectiveness of caplacizumab has been compared with a placebo plus standard of care and glucocorticoids in two double-blind trials, TITAN (2016) and HERCULES (2019) [8,9]. In the phase 2 TITAN trial, published in 2016, 75 adults with iTTP were randomized to receive caplacizumab 10 mg daily or a placebo for 30 days after TPE discontinuation. Exacerbation occurred in 28% of the patients in the placebo group and 8% in the caplacizumab group. However, after discontinuation of caplacizumab, eight patients (22%) of the intervention arm showed recurrent thrombocytopenia, seven of whom had persistent severe ADAMTS13 deficiency (none in the placebo arm). These episodes were nonetheless considered relapses rather than exacerbations since their onset was reported later than 30 days after TPE interruption, although within 30 days from caplacizumab interruption [8]. Successively, the HERCULES trial extended up to 28 additional days after TPE discontinuation of caplacizumab treatment in patients showing persistent, severe ADAMTS13 deficiency, while optimizing immunosuppressive therapy. The results highlighted a markedly decreased rate of relapses. Moreover, a lower incidence of the composite outcomes of iTTP-related death, recurrence of iTTP, or thromboembolic event was found in the caplacizumab arm compared to the placebo (12% vs. 49%; *p* < 0.001) [9]. In view of the positive results of these RCTs, between 2018 and 2019, caplacizumab was approved by the European Medicines Agency (EMA) and by the US Food and Drug Administration (FDA) for the initial treatment of iTTP in combination with standard-of-care [10,11]. In Italy, caplacizumab was approved for reimbursement to treat adults with iTTP in combination with TPE and immunosuppression on 17 January 2020 [12].

The data from clinical trials have been successively corroborated by real-world evidence (RWE) studies that confirmed the significant clinical and economic benefits of caplacizumab over traditional therapy [13,14].

To date, there is poor evidence in Italy on the epidemiology of iTTP. Moreover, since aTPP is a rare disease, data on rebounds of the introduction of caplacizumab treatment on large populations outside clinical trials are lacking.

This present real-world analysis primarily aimed at analyzing the number and length of hospitalizations and mortality rates among aTPP patients treated or untreated with caplacizumab. Secondly, the patients with and without caplacizumab were characterized by their general demographic features, comorbidity profile, and previous history of iTTP-related hospitalizations. Lastly, the trends in the annual incidence of iTTP in the 3-year period before and after caplacizumab introduction in Italy (2020) were also estimated.

## 2. Materials and Methods

### 2.1. Data Source

An observational retrospective analysis was performed using data retrieved from the administrative databases of samples of Italian healthcare entities covering nearly 17 million health-assisted individuals (corresponding to approximately 29% of the entire country’s population). The participating Local Health Units were selected by geographical distribution (by North/Center/South Italy), by data completeness, and by the high-quality linked datasets. The selected healthcare entities belonged to 11 Italian regions (i.e., Veneto, Piedmont, Lombardy, Liguria, Umbria, Lazio, Abruzzo, Molise, Apulia, Campania, and Sicily).

The data used for the analysis were extracted from the following databases: (i) demographic database—to collect information on patients’ demographic data, namely gender, age, and date of death (if applicable); (ii) pharmaceuticals database—for all the information on medicinal products reimbursed by the Italian National Health Service (NHS), namely the Anatomical Therapeutic Chemical (ATC) code, number of packages, number of units per package, unit cost per package, and date of prescription; (iii) hospitalization database—to obtain data on hospitalizations, like discharge diagnosis codes classified according to the International Classification of Diseases, Ninth Revision, Clinical Modification (ICD-9-CM), Diagnosis Related Group (DRG), and DRG-related charge (provided by the Italian NHS); (iv) outpatient specialist services database—to record data on specialist visits, laboratory testing, and diagnostic procedures, namely date and type of provision, description of the activity, and specialist visit charge; (v) exemption database—to gather information on the active payment waiver codes by which patients are discharged from paying services/treatments in case of specific disease diagnoses.

In order to ensure privacy, an anonymous univocal numeric code (patient ID) was given to each participant in the analysis. The patient ID allowed for the electronic linkage between databases and also warranted the anonymity of the extracted data in full compliance with UE Data Privacy Regulation 2016/679 (“GDPR”) and Italian D.lgs. n. 196/2003, as amended by D.lgs. n. 101/2018. All the findings were presented as aggregated data so that they could not identify, either directly or indirectly, individual patients. The analysis was conducted in line with the principles of the Declaration of Helsinki and approved by the local Ethics Committees of the participating healthcare entities.

### 2.2. Study Design and Patient Population

From a sample population of almost 17 million health-assisted individuals, hospitalized adult (≥18 years) patients with a diagnosis of iTTP were identified by the ICD-9-CM code 446.6 from January 2018 to the end of the data availability (up to March 2023) inclusion period. Women with complications of pregnancy identified by the codes ICD-9-CM 634–639 (other pregnancy with abortive outcome) and ICD-9-CM 640–649 (complications mainly related to pregnancy) were excluded from the analysis. Moreover, patients treated with caplacizumab were identified by the presence of at least one prescription for caplacizumab (ATC B01AX07). The group untreated with caplacizumab consisted of patients with iTTP without a prescription for caplacizumab by considering all of the available observational period. The index date was the time of the first prescription of caplacizumab for the treated cohort and the time of first hospitalization with iTTP discharge diagnosis (throughout the whole inclusion period) for the caplacizumab-untreated cohort. The characterization period was all the time of data availability preceding the index date, and the follow-up was all of the available period after the index date.

For all patients with iTTP, demographic variables in terms of age, distribution of subjects below or over 50 years of age, and gender (proportion of males) were recorded at inclusion. The comorbidity profile was assessed during the characterization period using the Charlson Comorbidity Index, a score resulting from the sum of weight assigned to 19 concomitant conditions; thus, 0 indicates no comorbid conditions, while higher scores indicate a greater level of comorbidity [15]. Moreover, the mean number and the mean duration of iTTP-related hospitalization before the index date (if any) were collected.

The primary outcomes of this analysis were the number and length of ordinary and ICU hospitalizations and mortality rates among patients treated or untreated with caplacizumab. As secondary endpoints, the groups were described for their general demographic features, comorbidity profile, and previous history of TTP-related hospitalizations. Lastly, the trends in the annual incidence of iTTP in the 3-year period before and after caplacizumab introduction in Italy (2020) were estimated.

### 2.3. Statistical Analysis

All the analyses were purely descriptive. Continuous variables are reported as mean ± standard deviation (SD) and median and interquartile range (IQR, 25° and 75° percentile); categorical variables are expressed as frequencies and percentages. Patients were divided into treated and untreated with caplacizumab. For the untreated cohort, a propensity score matching (PSM) was applied to select a subgroup of patients comparable with caplacizumab-treated ones for demographic age, clinical characteristics (i.e., Charlson comorbidity Index), and the mean number/patient of iTTP hospitalization prior the index date. All data were reported as descriptive for caplacizumab-treated cohort and caplacizumab-untreated cohorts, PSM matched and unmatched.

Following the ‘Opinion 05/2014 on Anonymization Techniques’ drafted by the ‘European Commission Article 29 Working Party’, the analyses involving fewer than three patients were not reported, as they were potentially traceable to single individuals. Therefore, the results referring to <4 patients were not reported and therein indicated as not issuable (NI). All the analyses were performed using STATA SE version 17.0 SE (StataCorp LLC, College Station, TX, USA).

## 3. Results

### 3.1. Annual Incidence of iTTP Hospitalization and Projection to the Italian Population

The annual incidence iTTP, defined as the annual number of patients hospitalized with iTTP diagnosis (per 1,000,000 people), was estimated during a 6-year time horizon, covering 3 years before (2017, 2018, 2019) and after (2020, 2021, 2022) caplacizumab introduction into the Italian market. The incidence of iTTP prior to caplacizumab introduction was 4.3 cases/million in 2017, 4.5 cases/million in 2018, and 5.8 cases/million in 2019; instead, after the introduction of caplacizumab, there were 4.4 cases/million in 2020, 3.6 cases/million in 2021, and 4.6/million in 2022.

### 3.2. Baseline Characteristics and Outcomes in iTTP Patients Treated and Untreated with Caplacizumab before and after Propensity Score Matching

Across 2018–2022, overall, 393 patients with iTTP were included in this study, of whom 42 received caplacizumab. The baseline characteristics and clinical outcomes were investigated in iTTP patients treated and untreated with caplacizumab before and after applying a PSM with a 1:4 ratio (between iTTP patients treated and untreated with caplacizumab, respectively). The PSM allowed for balancing the baseline characteristics of 168 untreated patients with 42 patients treated with caplacizumab.

Table 1 depicts the main demographic and clinical characteristics in caplacizumab-treated patients and those untreated before and after PSM.

Considering the patients treated with caplacizumab, the age at the index date (first prescription of caplacizumab) averaged 46.8 ± 11.4 years (median age: 48.5 years), and 31% were males. The comorbidity profile was in general mild, as documented by a mean (±SD) Charlson Comorbidity Index of 0.6 ± 0.8 (median: 0.0). In this cohort, the mean (±SD) number of previous iTTP hospitalizations of 0.9 ± 1.7 (median: 0.0) per patient and a mean (±SD) duration of previous iTTP hospitalizations (calculated only in previously hospitalized patients) of 15.8 ± 12.0 days (median: 12.5 days) were found during all of the available period before caplacizumab therapy initiation (mean duration 9.5 ± 3.7 years).

Among the raw, unadjusted cohort of caplacizumab-treated patients (*N* = 351), the mean age was 57.6 years (median: 58.0 years), the Charlson Comorbidity Index averaged 1.6 (median: 1.0), and the mean number/patient of previous iTTP hospitalizations was 0.4 (median: 0.0), with a mean duration of 54.1 2 ± 74.3 days (median: 20.0 days).

After PSM-balancing for age and Charlson Comorbidity Index, the untreated patients (*N* = 168) showed a mean (±SD) number of previous iTTP hospitalizations of 0.7 ± 2.0 (median: 0.0) per patient, with a mean (±SD) duration of 53.2 ± 73.6 days (median: 20.0 days).

Table 2 depicts the main clinical endpoints evaluated during the follow-up in caplacizumab-treated patients and those untreated before and after PSM.

In patients treated with caplacizumab, no deaths (0/42) were recorded after 1 month and 3 months from the first prescription, and the mean duration of hospital stay in the ordinary setting (for any cause and evaluated up to one-year follow-up) was 15.7 ± 11.1 days (median: 13.0 days). Fewer than four patients (<9.5%) were admitted to ICU during the first year of follow-up (including the index date), thus not allowing to report the mean duration of ICU hospitalizations due to data privacy reasons (sample size < four patients). The overall duration of the ICU hospitalizations was <4 days. By considering the entire follow-up period available (mean: 13.5 months), the number of caplacizumab vials prescribed averaged 31.2 ± 20.3 per patient (median: 29.0 vials).

In the untreated patients before PSM balancing, the mortality rate 1 month after the index date was 10.5%, while at 3 months it was 11.1%. The mean duration of ordinary hospitalization was 29.0 ± 45.49 days (median: 16.0 days), while the mean duration of ICU hospitalization was 10.5 ± 13.2 days (median: 6.0 days; 408 days in total during the mean follow-up of 26.2 months).

In the PSM-adjusted cohort of untreated patients (*N* = 168), the mortality rate was 8.9% and 10.1% at 1 and 3 months, respectively. The mean duration of ordinary hospitalization was 30.3 ± 44.4 days (median: 15.0 days), and the mean duration of ICU hospitalization was 9.4 ± 9.4 days (median: 6.0 days; 207 days in total during the mean follow-up of 28.5 months).

## 4. Discussion

This analysis was undertaken primarily to fill the informative gap about the role of the caplacizumab introduction into the daily clinical practice in Italy, and secondarily to assess the iTTP epidemiology data and characteristics of patients.

The current real-world analysis showed that at baseline, patients treated with caplacizumab were relatively young (on average 46.8 years) and predominantly females. This population mirrored the data of existing literature, coming from both RCT analyses and European RWE, indicating that iTTP patients on caplacizumab therapy were aged 45–46 years, and about two-thirds were women [9,13,14].

The analysis of main clinical endpoints, such as all-cause mortality, hospitalizations, and ICU admissions, revealed that no patient who received caplacizumab died within the short-term follow-up of 1 and 3 months, in front of mortality rates of around 10–11% among those on standard therapy at the same time points. Although the small sample size of the caplacizumab cohort allowed us to present purely descriptive data, a trend towards better clinical outcomes seems to emerge in caplacizumab-treated patients, in terms of a lower likelihood of being hospitalized for iTTP and requiring ICU access and in general shortened hospital stays, as compared to the untreated ones. Nonetheless, it should also be noticed that the two cohorts were different in age and clinical status since those on standard therapy only were older (57.6 years, compared to 46.8 years of caplacizumab-treated patients) and had a more than doubled Charlson index (1.6 compared to 0.6 of caplacizumab-treated patients). To minimize selection bias due to these differences, the groups were further analyzed after PSM balancing. However, after matching the groups with or without caplacizumab for baseline covariates, the post-PSM analysis confirmed the results on main clinical endpoints, as caplacizumab-treated patients still had tendentially more favorable outcomes in terms of all-cause mortality and iTTP-related hospitalizations. These data are in line with the clinical trial HERCULES [9] and other European RWE analyses [13,14]. In the HERCULES trial, where iTTP-related death was assessed during the treatment period (30 days), the iTTP-related mortality of caplacizumab-treated patients was 0% (0/72), compared to 4% (3/73) in the placebo group [9]. Data from a French observational analysis showed 1.1% mortality within 30 days since diagnosis in patients treated with caplacizumab and 6.7% mortality among historical iTTP cohort included before the introduction of caplacizumab into French clinical practice [16]. Moreover, a recent Italian retrospective analysis carried out by using the Milan TTP Registry reported that among 26 patients treated with caplacizumab for an acute iTTP episode, all patients had a remission with 0% mortality rate [17,18].

Concerning the hospitalization rates and length, our findings are also corroborated by previous evidence from the literature. Coppo et al. showed a median length of hospitalization of 13 days and 22 days for the cohort treated with caplacizumab and the historically untreated cohorts, respectively [19]. These results were also confirmed by Izquierdo et al., who reported a statistically significant difference in hospitalization days between patients treated and untreated with caplacizumab (12 days vs. 19 days, respectively, *p* < 0.001) [12]. Moreover, Völker et al. described a mean hospitalization length of 21.6 days in patients treated with caplacizumab [14]. Similarly, the analysis of the Milan TTP Registry indicated a median length of hospital stays for patients treated with caplacizumab of 18 days [17]. The therapy with caplacizumab implied a mean consumption of around 31 vials per patient during the overall observation period; data from the Milan Registry and German RWE analyses showed a median duration of caplacizumab treatment of 26 and 34 days, respectively [14,17,18].

In our sample covering approximately 29% of the entire Italian population and belonging to 11 Italian regions, the mean yearly incidence of patients hospitalized for iTTP was 4.5 cases per million people, with an almost linear increase trend across 2017–2022. Moreover, the overall 2017–2022 increasing trend observed in iTTP diagnoses might be explained by several educational activities started across the country among interprofessional teams for improving care coordination and communication to advance the diagnosis of iTTP. The epidemiological data on iTTP reported in this study are in agreement with the Italian data previously published by the Istituto Superiore di Sanità (ISS) [20,21]. Moreover, the incidence in Europe has been estimated to be between 1.5 and 6.0 cases per million [16,22,23,24,25], consistent with the current report.

Although cost analysis was not among the aims of this present investigation, some points deserve further reflection. Caplacizumab was proven to be a successful option in treating iTTP, so it is regarded as a potentially life-saving agent. However, the elevated cost remains a major barrier to its widespread utilization. Anyhow, according to recent evidence, the cost-effectiveness of caplacizumab relies on several concurrent conditions, including the requirement of rituximab and TPE, hospitalizations, and intensive care stays [26]. Real-world data from 29 German medical centers confirmed caplacizumab effectiveness for iTTP treatment, independent of timing or therapy modalities, and suggested that earlier use of caplacizumab reduced the need for TPE [27]. Moreover, using ADAMTS13 to guide the duration of therapy allowed 58.3% of patients to be treated with caplacizumab for less than 30 days, thus sparing caplacizumab vials and resulting in cost savings of EUR 2.49 million [28].

The present results must be interpreted considering some current limitations related to the observational nature of the analysis and the use of data extracted from administrative databases. The analysis was carried out among a sample corresponding to 29% of the entire Italian population, belonging to 11 regions and geographically distributed across the national territory. Thus, since the sample covers a limited number of healthcare entities, the results might suffer from uncaptured diversities in clinical practice settings. Moreover, one intrinsic flaw of administrative databases is that they are not originally meant to be used for research purposes, and thus certain events and diagnoses might be incomplete or lacking. Administrative data are collected for reimbursement, not for coordinating medical care or conducting outcomes research, which could translate into incomplete information on disease severity, comorbidities, and other potential confounders that could have influenced the results. For instance, the Charlson Comorbidity Index was calculated using drug prescription and hospitalizations as proxies of diagnosis of each concomitant disease; therefore, untreated or non-hospitalized comorbidities were not captured. For this reason, in the analysis, a control cohort of patients untreated with caplacizumab was considered to overcome this bias, so uncaptured clinical features (e.g., the baseline clinical manifestations and baseline laboratory parameters) that could have influenced the outcome results would affect both groups. In fact, for data interpretation, it should be considered that some clinical variables, such as the levels of ADAMTS13 and bleeding complications, were not captured among administrative databases.

Moreover, it has to be acknowledged that the data presented here are purely descriptive rather than the results of a randomized study, and no comparative analyses were presented between patients treated with caplacizumab and untreated ones. This choice was made in view of the small sample size of the group of patients on caplacizumab therapy, which prevented us from conducting a case-control study or any comparative analysis. The population of patients with iTTP was filtered from the health records using the ICD-9-CM classification diagnostic code, as previously reported [29]; thus, if patients were not classified as such in their health records with an established iTTP diagnosis, they were not included in the analysis.

Despite these shortcomings, administrative data have been widely used and have been generally successful in evaluating the association between disease conditions and clinical/economic outcomes. Another strength is represented by the possibility of investigating unselected populations in real-life settings, thus comprising patients (as elderly or with a multimorbid profile) who are generally underrepresented in randomized clinical trials.

## 5. Conclusions

In conclusion, with respect to the previous retrospective analyses carried out by using data from the Milan TTP Registry collected from a unique center of excellence [17,18,30], this is the first real-world analysis of iTTP from several healthcare districts in Italy after the introduction of caplacizumab into the clinical practice. Such an approach could represent a powerful and necessary tool to systematically collect epidemiologic, clinical, and laboratory data with good representativeness of the Italian clinical practice. Therefore, this real-world analysis provided an up-to-date description of the current management of patients with iTTP, the results of treatments, as well as its epidemiology.

## Figures and Tables

**Table 1:** Baseline characteristics of iTTP patients treated and untreated with caplacizumab, before and after PSM.

	Caplacizumab-treated Patients (*N* = 42)	Caplacizumab-untreated Patients, Pre-PSM (*N* = 351)	Caplacizumab-untreated Patients, Post-PSM (*N* = 168)
Male gender*, N* (%)	13 (31.0%)	156 (44.4%)	53 (31.5%)
Age at index date, years, mean (SD)	46.8 (11.4)	57.6 (17.5)	47.5 (15.0)
Age at index date, years, median (IQR)	48.5 (40.2–53.8)	58.0 (45.5–71.0)	48.5 (36.0–57.0)
Age, years, min–max	(19–69)	(18–96)	(18–83)
Age < 50 years*, N* (%)	24 (57.1%)	115 (32.8%)	90 (53.6%)
Age ≥ 50 years*, N* (%)	18 (42.9%)	236 (67.2%)	78 (46.4%)
Charlson Comorbidity Index, mean (SD)	0.6 (0.8)	1.6 (1.9)	0.6 (0.8)
Charlson Comorbidity Index, median (IQR)	0.0 (0.0–1.0)	1.0 (0.0–2.0)	0.0 (0.0–1.0)
Charlson Comorbidity Index = 0, *N* (%)	23 (54.8%)	112 (31.9%)	99 (58.9%)
Charlson Comorbidity Index ≥ 1, *N* (%)	19 (45.2%)	239 (68.1%)	69 (41.1%)
Number of previous iTTP hospitalizations, mean (SD)	0.9 (1.7)	0.4 (1.5)	0.7 (2.0)
Number of previous iTTP hospitalizations, median (IQR)	0.0 (0.0–1.0)	0.0 (0.0–0.0)	0.0 (0.0–0.0)
Length of previous iTTP hospitalization, days, mean (SD)	15.8 (12.0)	54.1 (74.3)	53.2 (73.6)
Length of previous iTTP hospitalization, days, median (IQR)	12.5 (9.0–16.5)	20.0 (11.8–58.0)	20.0 (12.0–54.0)
Length of available characterization period, years, mean (SD)	9.5 (3.7)	8.7 (4.0)	8.0 (3.9)

Abbreviations: IQR, interquartile range (25° and 75° percentile); SD, standard deviation.

**Table 2:** Descriptive analysis of the clinical outcomes in iTTP patients treated and untreated with caplacizumab, before and after PSM.

	Caplacizumab-treated Patients (*N* = 42)	Caplacizumab-untreated Patients, Pre-PSM (*N* = 351)	Caplacizumab-untreated Patients, after PSM (*N* = 168)
*Outcomes*			
Mortality at 1 month (after index date), *N* (%)	0 (0.0%)	37 (10.5%)	15 (8.9%)
Mortality at 3 months (after index date), *N* (%)	0 (0.0%)	39 (11.1%)	17 (10.1%)
Length of all-cause ordinary hospitalization *, days, mean (SD)	15.7 (11.1)	29.0 (45.9)	30.3 (44.4)
Length of all-cause ordinary hospitalization *, days, median (IQR)	13.0 (9.0–17.0)	16.0 (7.0–28.0)	15.0 (7.0–30.0)
Patients hospitalized in ICU *, *N* (%)	<4 (<9.5%)	39 (11.1%)	22 (13.1%)
Length of ICU hospitalization *, days, mean (SD)	NI	10.5 (13.2)	9.4 (9.4)
Length of ICU hospitalization *, days, median (IQR)	NI	6.0 (1.0–15.0)	6.0 (2.2–17.0)
Overall duration of ICU hospitalization (all patients), days	<4	408	207
Number/patients of caplacizumab vials prescribed ^§^, mean (SD)	31.2 (20.3)	-	-
Number/patients of caplacizumab vials prescribed ^§^, median (IQR)	29.0 (14.2–39.8)		
Follow-up, months, mean	13.5	26.2	28.5

* up to one year after the index date; ^§^ overall period. Abbreviations: ICU, intensive care unit; IQR, interquartile range (25° and 75° percentile); NI, not issuable; PSM, propensity score matching; SD, standard deviation.

## Data Availability

All data used for the current study are available upon reasonable request by CliCon s.r.l., which is the body entitled of data treatment and analysis by the healthcare entities involved in this study.
